# Outcomes of Non-operatively Managed Achilles Tendon Rupture: A Retrospective, Single-Centre Study

**DOI:** 10.7759/cureus.100235

**Published:** 2025-12-28

**Authors:** Adam Daneshyar, Sajid Ali, Mohanraj Venkatesan, Omar Mostafa, Anam Jawaid, Qutub Qadri, Kowshik Jain

**Affiliations:** 1 Trauma and Orthopaedics, Dudley Group NHS Foundation Trust, Birmingham, GBR

**Keywords:** achilles rupture, achilles tendon, achilles tendon (at), achilles tendon injury, achilles tendon rehabilitation, conservative management of achilles tendon

## Abstract

Introduction

Achilles tendon ruptures can be managed surgically or conservatively. Due to the increased risk of post-operative complications, non-surgical management is often preferred. Evidence suggests that non-operative approaches can achieve outcomes comparable to surgical repair. One such approach is the Leicester Achilles Management Protocol (LAMP), which involves immobilisation in a VACOped boot for eight weeks. Patient outcomes following non-operative management are commonly assessed using the Achilles Tendon Total Rupture Score (ATRS). In this retrospective study, it is hypothesised that patients managed with the LAMP protocol will report favourable outcomes as measured by the ATRS.

Methodology

This retrospective cohort study analysed patients who presented and were managed for Achilles tendon rupture at a district general hospital in the West Midlands, UK, between January 2022 and October 2024. Patients aged 18 years and above with confirmed Achilles tendon injuries (International Classification of Diseases, Tenth Revision (ICD-10) code: S86.0), managed through fracture or elective clinics, were included in the analysis. Data obtained from electronic health records included demographics, comorbidities, management duration, time to rehabilitation, and patient-reported outcomes using the ATRS. ATRS data were collected via telephone interviews conducted by local clinicians and analysed descriptively using frequencies, means, and ranges.

Results

A total of 53 patients were initially screened for inclusion. Eight patients were excluded following clinic reassessment due to misdiagnosis (no rupture or posterior tibial rupture) or death. Of the remaining 45 patients contacted by telephone, 16 did not respond after two attempts, and one declined participation. The final study cohort comprised 28 patients.

The analysis showed that patients were mainly male, with an average age of 52.1 years (range: 29-82). Patient-reported outcome measures showed the mean ATRS was 67.1 (range: 26-100), reflecting a wide variation in self-reported recovery.

Conclusion

Despite a lower ATRS compared to previous studies with non-operative management, this retrospective study showed that LAMP provides satisfactory functional recovery whilst avoiding operative and post-operative complications.

## Introduction

The Achilles tendon, the largest and strongest tendon in the body, lies in the superficial posterior compartment of the leg and connects the gastrocnemius and soleus muscles to the calcaneus. Despite its strength, it is susceptible to degenerative changes that increase the risk of rupture, particularly with advancing age, systemic disease, certain medications, overuse, impaired vascularity, or pre-existing tendinopathy.

Management of Achilles tendon rupture can be surgical or conservative. Surgery is generally indicated for delayed presentations (more than two to three weeks), re-ruptures, avulsion injuries, or cases with fatty tissue filling the tendon gap. While operative repair reduces re-rupture risk, it is associated with higher rates of minor complications compared with non-operative treatment, such as wound problems, swelling, bruising, temporary nerve irritation, stiffness, scar tenderness, small hematomas, and delayed tendon healing [1].

Recent meta-analyses [2,3] have shown that non-operative management can achieve comparable functional outcomes and high patient satisfaction, especially when protocols emphasise early mobilisation and progressive loading in comparison to the surgical approach. Conservative treatment avoids surgical risks, making it suitable for patients with co-morbidities such as diabetes, cardiovascular disease, peripheral vascular disease, or for those preferring to avoid surgery, although re-rupture rates may be slightly higher.

Non-operative management is commonly guided by the Leicester Achilles Management Protocol (LAMP), which involves immobilisation in a VACOped boot for eight weeks. The boot is initially set at 30° of plantarflexion for four weeks, followed by progressive adjustment over two weeks, and finally a wider range of motion from neutral to 30° of plantarflexion during the last phase. Continuous use is advised, with removal only for hygiene purposes. Conservative treatment carries recognised risks, including re-rupture within the first three months, calf weakness, and venous thromboembolism.

Patient-reported outcome measures (PROMs) have become central to evaluating recovery following Achilles tendon rupture, as they capture the patient’s perspective on symptoms, function, and overall quality of life. The Achilles Tendon Total Rupture Score (ATRS) is the most widely used tool. It consists of 10 items addressing symptoms such as pain, stiffness, and weakness, as well as activity-related limitations including walking, running, and participation in sport [4]. Scores range from 0 to 100, with higher scores reflecting better function. While other tools, such as the Victorian Institute of Sports Assessment - Achilles (VISA-A), exist, evidence suggests that the ATRS is more responsive and better aligned with rupture-specific recovery, making it the preferred PROM in both clinical practice and research [5].

Given the rising incidence of Achilles tendon ruptures in the UK, which increased from six to 13 per 100,000 person-years between 1995 and 2019 [6], and their associated impact on function, there is a need for contemporary data describing patient-reported outcomes following non-operative management.

The primary objective of this study was to descriptively evaluate patient-reported functional outcomes following non-operative management of acute Achilles tendon rupture using the LAMP, as measured by the ATRS. ATRS data were collected at a single time point, providing a cross-sectional assessment of patient-reported function at variable stages of recovery. Secondary objectives included describing variability in recovery and contextualising outcomes within the existing literature.

It is anticipated that patients managed using the LAMP protocol will report positive patient-reported outcomes using ATRS, including effective functional recovery, low pain, and high overall satisfaction.

## Materials and methods

This retrospective cohort study followed the STROBE (Strengthening the Reporting of Observational Studies in Epidemiology) guidelines and was conducted at the Dudley Group NHS Foundation Trust, West Midlands, United Kingdom. The study included all patients who presented with and were managed for Achilles tendon rupture between January 2022 and October 2024.

Study population

All adult patients aged 18 years or older who sustained an Achilles tendon injury and were managed through fracture or elective orthopaedic clinics were eligible for inclusion. Cases were identified using the International Classification of Diseases, Tenth Revision (ICD-10) code S86.0 (“Injury of Achilles Tendon”).

Inclusion criteria were patients aged ≥18 years with a confirmed diagnosis of acute Achilles tendon rupture based on clinical assessment and/or ultrasound imaging. All identified records were screened to confirm diagnostic accuracy and eligibility for inclusion in the final dataset (see Results).

Data collection and variables

Clinical information was retrieved from Sunrise electronic health records, clinic letters, and PROMs. With permission from the original publisher, the ATRS was used to assess patient-reported outcomes. ATRS data were collected and compiled on Excel (Microsoft Corporation, Redmond, WA) for the purpose of this study via a single round of retrospective telephone interviews conducted by local clinicians in April 2025. Consequently, patients were assessed at differing intervals following injury depending on the year of rupture. While no formal inter-rater training or standardisation was conducted, all interviewers followed the same protocol for administering the questionnaire.

A retrospective consecutive sampling approach was used, including all eligible cases within the study period.

Collected variables included diagnostic confirmation (clinical and/or ultrasound), patient demographics, comorbidities, smoking status, duration of management time to commencement of rehabilitation, and ATRS outcomes. Potential confounding factors such as patient age, comorbidities, smoking status, and delay in physiotherapy initiation were identified and described descriptively but not statistically adjusted for.

Statistical analysis

Data were analysed using descriptive (frequency) statistics, including means, standard deviations, ranges, and proportions. All proportions are reported in the format of n (%), where n represents the absolute frequency. Given the exploratory design and limited sample size, inferential analyses, including confidence intervals and subgroup comparisons, were not undertaken to avoid overinterpretation.

Ethics

This study analysed retrospective anonymised data and therefore did not require formal ethics approval under local governance policy. However, institutional approval and audit registration were obtained from The Dudley NHS Foundation Group Audit Department (Audit ID: T&O/SE/2024-25/17).

## Results

**Figure 1:**
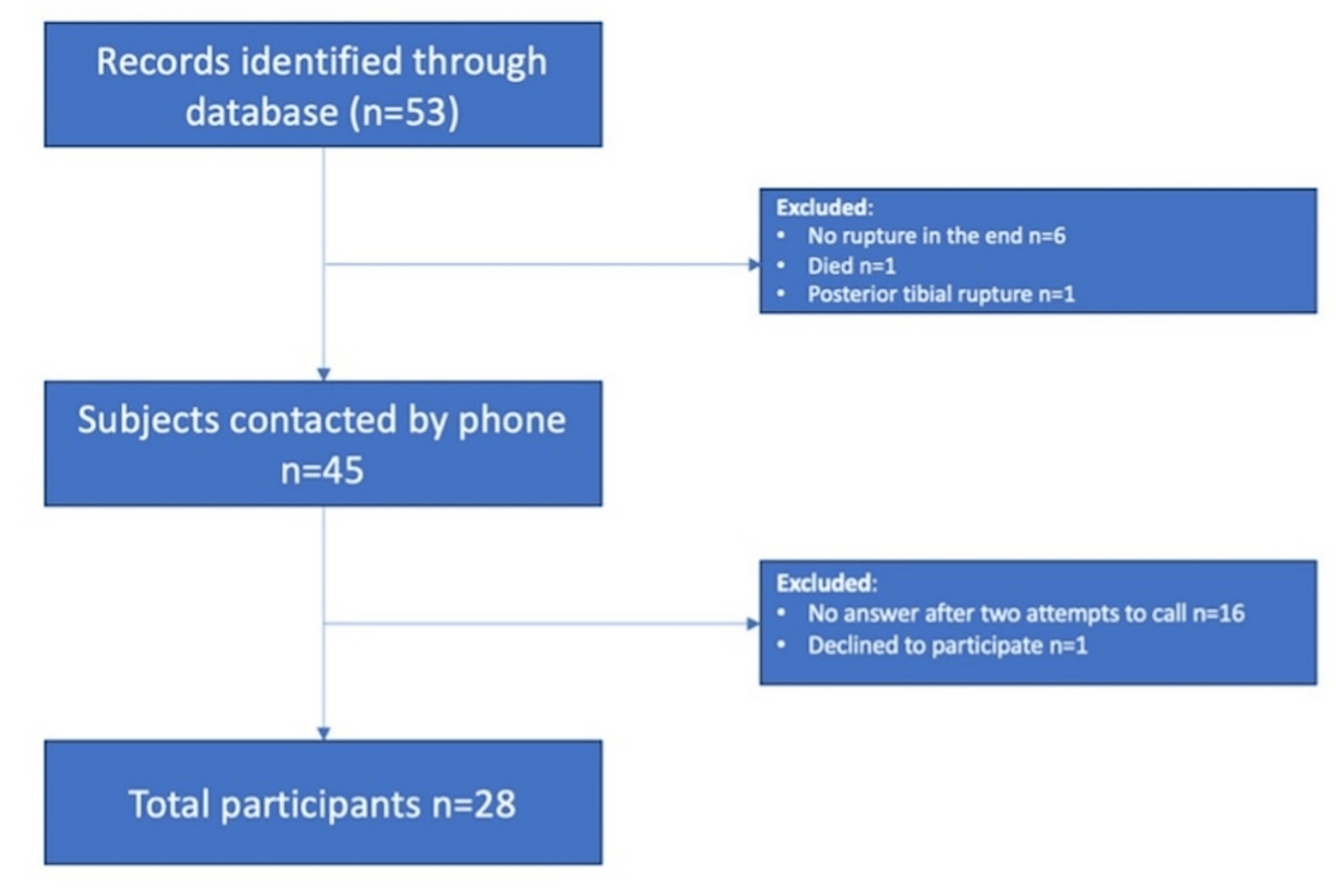
Flowchart illustrating the selection of eligible patients for the study.

A total of 53 patients were initially screened for the study. Before patient contact, eight (15.1%) patients were excluded (Figure 1); six (11.3%) due to an incorrect initial diagnosis with no Achilles tendon rupture, one (1.9%) with a posterior tibial tendon rupture, and one (1.9%) who had died following the rupture.

The remaining 45 (84.9%) patients were contacted by local doctors at the hospital. A total of 16 (35.6%) patients failed to answer after two attempts, and one (2.22%) patient declined to participate for an unknown reason. Therefore, 28 (62.22%) patients were included in this study.

The mean age of the cohort was 52.1 years (range: 29-82), with a predominance of male patients (20, 71.4%). The prevalence of co-morbidities was as follows: two (7.1%) patients had diabetes, three (10.7%) had a documented cardiac history, and one (3.6%) was a current smoker. The majority of cases represented a first-time rupture (25, 89.3%) (Table 1).

**Table 1:** Demographics of eligible patients.

Demographics	
Average age (years)	52.1
Male	20 (71.4%)
Female	8 (28.6%)
Diabetes mellitus	2 (7.14%)
Cardiac history	3 (10.71%)
Smoking	1 (3.6%)
First rupture	25 (89.3%)

The mean time from injury to initial orthopaedic review was 5.5 days (range: 1-12). Ultrasound imaging was performed in eight (28.6%) cases, with an average delay of 12.9 days between referral and completion.

Management was predominantly conservative (26, 92.9%), with one (3.6%) patient undergoing surgical repair and a further one patient (3.6%) awaiting surgery at the time of data collection. Patients attended a mean of 3.2 outpatient appointments (range: 2-5) and were followed up for 8.8 weeks on average (range: 7-12) before discharge from routine care.

All patients were enrolled in a structured rehabilitation programme. The average weeks of follow-up prior to discharge in the clinic averaged 8.6 weeks. The first physiotherapy appointment occurred a mean of 13.3 days after being discharged from the clinic (range: 2-25).

PROMs were captured using the ATRS. Among respondents, the mean ATRS was 67.1 (range: 26-100), reflecting a wide variation in self-reported recovery. As all ATRS scores were obtained during a single data collection period, ATRS values were further analysed by year of injury to examine cohort variation (Table 2).

**Table 2:** Mean ATRS scores grouped by year of injury. ATRS: Achilles Tendon Total Rupture Score.

Injury year (date range)	Number of patients (n)	Mean ATRS
09/01/2022 - 31/12/2022	5	79.2
01/01/2023 - 31/12/2023	15	68.1
01/01/2024 - 31/10/2024	8	57.5

## Discussion

The results demonstrated a mean ATRS score of 67.1, indicating satisfactory functional outcomes. These findings contribute to the growing body of evidence that non-operative approaches with early functional rehabilitation can achieve recovery outcomes comparable to surgical repair while avoiding operative risks [1,2].

Compared with published data, where mean ATRS scores typically range between 81 and 82 at one-year follow-up [7,8], our study has a considerably lower score. Aujla et al. [9] report a mean ATRS of 75.5 in a cohort of 234 patients managed with the LAMP protocol. The wide range of scores in our cohort (26-100) demonstrated that recovery varied considerably between patients. Several factors could have contributed to the lower and varied ATRS score, such as delayed presentation, differences in adherence to rehabilitation, and variation in baseline activity levels or expectations. The lower ATRS scores compared to previously mentioned studies should be viewed as reflecting variability in individual recovery rather than a failure of non-operative management. Although the mean ATRS was 67.1, most patients regained adequate function for daily activities without the need for surgery.

Access to physiotherapy occurred on average 13.3 days after being discharged from the clinic. There is no specific timeline for initiating physiotherapy within LAMP; however, evidence supports that early functional rehabilitation enhances tendon healing through controlled loading and reduces re-rupture risk compared with prolonged immobilisation [10]. Given the wide range (2-25 days) of starting physiotherapy after clinic, the later initiation for some patients may have negatively influenced early functional recovery. Additionally, adherence to the LAMP protocol outside supervised sessions was not measured in this study, representing a potential unaccounted confounder that may have contributed to variability in outcomes.

ATRS data were collected via telephone, which may contribute to lower scores due to recall bias, limited opportunity for clarification or demonstration of function, and variability in patient interpretation of questions. Collectively, these factors likely contributed to the lower mean ATRS observed in this study compared with previous reports.

All ATRS scores were collected at a single time point, meaning patients were assessed at different stages of recovery depending on the year of injury. While stratification by injury year showed lower mean ATRS scores in more recently injured patients, this is most likely due to shorter recovery time rather than genuine differences in functional outcome between groups. As a result, direct comparisons between cohorts are limited, and the overall mean ATRS is likely underestimated because of variability in follow-up duration. Given that PROMs such as the ATRS are time-dependent, these results should be interpreted descriptively rather than as a reflection of the recovery trajectory. Future studies should use standardised follow-up intervals for data collection to allow more meaningful comparison of functional outcomes over time.

The study cohort generally reflected the population most commonly affected by Achilles tendon rupture, with a mean age of 52 years and a predominance of male participants. This aligns with recent epidemiological data showing that middle-aged men are at higher risk. The cohort also had a low prevalence of comorbidities, such as diabetes and cardiac disease, which is typical for this type of injury [6].

Male patients in this study demonstrated a higher ATRS score (71.3) compared with female patients (56.6), suggesting a trend towards superior self-reported functional outcomes. This observation is consistent with previous literature indicating that men often recover more rapidly and achieve higher levels of physical function following Achilles tendon rupture [9,11]. Potential explanations for this disparity include differences in baseline muscle mass, differing activity levels and tendon loading patterns [12]. In contrast, female patients may experience relatively slower tendon healing, potentially influenced by hormonal modulation of collagen synthesis and tendon remodelling. Studies suggest that women have lower rates of tendon collagen synthesis compared with men [13], particularly during certain phases of the menstrual cycle, which may affect recovery after tendon injury.

One of the main limitations of this study was the small sample size, with only 28 patients completing the ATRS, which may introduce non-response bias as the outcomes of patients who did not respond to follow-up may differ systematically from those who participated. As a single-centre, retrospective study, the analysis aimed to describe real-world trends in PROMs following non-operative management using the LAMP protocol. While the limited cohort restricts generalisability, focusing on descriptive outcomes remains appropriate for an exploratory analysis. Descriptive statistics allow meaningful interpretation of trends and variability in PROMs, rehabilitation timing, and functional recovery.

In addition, important prognostic variables such as tendon gap size, injury chronicity, and baseline activity level were not consistently available. Despite the limitations, the data provide valuable real-world insights into non-operative management with the LAMP protocol and contribute to the growing evidence supporting conservative treatment of Achilles tendon rupture. The findings demonstrate that, with appropriate immobilisation, early rehabilitation, and patient engagement, non-operative treatment can achieve satisfactory functional outcomes and provide a foundation for future, larger multi-centre study.

## Conclusions

Although the mean ATRS in our cohort was lower than that reported in previous LAMP studies, these findings suggest that non-operative management using the LAMP protocol can facilitate satisfactory functional recovery while avoiding operative risks. However, interpretation of these results must be cautious, as a proportion of eligible patients did not contribute PROM data, representing a potential source of non-response bias. Future prospective studies with standardised follow-up intervals and improved PROM completion rates are required to more robustly evaluate functional outcomes following non-operative Achilles tendon rupture management.
